# A Novel Scoring System for Assessing In-Hospital Mortality Risk in Patients With Liver Cirrhosis

**DOI:** 10.7759/cureus.72823

**Published:** 2024-11-01

**Authors:** Ni Luh Putu Yunia Dewi, I Ketut Mariadi, Kadek Mercu Narapati Pamungkas, Putu Itta Sandi Lesmana Dewi, Ni Nyoman Gita Kharisma Dewi, Dwijo A Sindhughosa

**Affiliations:** 1 Gastroenterology and Hepatology, Centre Research for Alimentary and Hepatobiliary System, Denpasar, IDN; 2 Gastroenterology and Hepatology, Internal Medicine, Udayana University, Prof. Dr. I.G.N.G. Ngoerah Central General Hospital, Denpasar, IDN

**Keywords:** in-hospital mortality, liver cirrhosis, prognosis, scoring system, viral hepatitis b and c

## Abstract

Background

Mortality among patients with liver cirrhosis has recently increased in Indonesia. However, predicting the prognosis of patients hospitalized with liver cirrhosis remains a clinical challenge due to its variability and dependence on multiple factors. A simple and accurate method is required to identify high-risk patients. This study aims to build a predictive scoring system of in-hospital mortality in patients hospitalized with liver cirrhosis for clinical application.

Methods

A retrospective cohort study was done to collect data on patients with liver cirrhosis from November 2021 to January 2022. The study involved 110 patients hospitalized with liver cirrhosis. A multivariate regression analysis was performed to identify factors predicting in-hospital mortality. Each variable's score was determined by applying the (B/SE)/lowest B/SE formula. The overall probability was calculated using the equation 1/1+exp(-y). Analysis of area under the curve (AUC) was conducted to evaluate the sensitivity and specificity of the scoring system.

Results

A total of 52 patients (47.3%) died during hospitalization. The median age of the patient was 54.5 (30-82). A final model involving the presence of hepatic encephalopathy (HE) (p = 0.001), ascites (p = 0.025), diabetes mellitus type II (p = 0.003), acute kidney injury (p = 0.017), alanine transaminase (ALT) ≥ 68 (p = 0.001), creatinine level ≥ 1.25 (p = 0.011), and abnormal international normalized ratio (INR) (p = 0.047). The “ADRECIA” score was developed, consisting of ascites, type II diabetes mellitus, renal injury, hepatic encephalopathy, creatinine serum, INR, and ALT. The presence of HE and ALT ≥68 was scored as two, and the rest variables were scored as one. The best-discriminating value was at a cut-off point ≥ 2.5, with a sensitivity of 90.4%, and a specificity of 74.1%, and an AUC of 0.913 (95%CI: 0.862-0.964). The scoring system was categorized as low risk (score of zero to three) with a 1.4-43.6% probability of death and high risk (score of 4-9) with 74.7-99.9% probability of death.

Conclusion

This scoring system provides good accuracy in predicting in-hospital mortality in patients with liver cirrhosis. Therefore, treatment can be modified according to the score to reduce mortality rates.

## Introduction

Liver cirrhosis is a non-malignant disease and is frequently a leading cause of death. Global mortality due to liver cirrhosis and chronic liver disease increased by 15% during the 2007-2017 period. The incidence of liver cirrhosis also increased in the Asia Pacific region following 2000-2015, including Indonesia [1]. In 2017, there were more than 1.32 million cirrhosis-related deaths, which contributed to 2.4% of total deaths globally. Southeast Asia has the fifth highest age-standardized cirrhosis-related death in the world [2]. In the last decade, Indonesia reported a total cirrhosis-related mortality of 49.224 people, which continued to increase between 1980 and 2010 [3].

Intrahepatic and extrahepatic factors can cause death in liver cirrhosis patients. Mortality in cirrhosis patients may be caused by complications such as hepatorenal syndrome, hepatic encephalopathy, or upper gastrointestinal bleeding [4]. Liver-related mortality is also influenced by age at diagnosis, presence of comorbidities, and severity of the disease [5]. A study conducted in a central hospital in Indonesia reported that infection was the most common cause of death in cirrhosis patients [6]. The poor survival rate of liver cirrhosis patients encouraged clinicians to explore more efficient treatments and find accurate prognostic evaluations to recognize and manage patients at high risk.

Existing clinical scoring systems, such as the Child-Turcotte-Pugh (CTP), model of end-stage liver disease (MELD), and MELD-Na, are effective in accurately predicting mortality at three and six months [7,8]. However, these scoring systems are less effective in predicting in-hospital mortality. The accuracy of MELD-Na in predicting hospital mortality of intensive care liver cirrhosis patients was 77%-81% [9]. A recent study reported accuracy of MELD in predicting hospital mortality in the Indonesian population was 82.6% [10]. One study in Indonesia also reported that the CTP score is not very effective in short-term mortality by showing a non-significant survival in CTP B compared to CTP C [11].

A study in Ghana by Duah et al. reported that hepatic encephalopathy, elevated international normalized ratio (INR), elevated MELD-Na, and creatinine level are predictors of in-hospital mortality. This finding implies that advanced disease and multiorgan dysfunction are associated with mortality [12]. A scoring system, referred to as the Chronic Liver Failure-Acute Decompensation (CLIF-AD), has been created to assess short-term and long-term mortality risks in individuals with decompensated cirrhosis. However, the scoring system has not been validated in Indonesia [13]. Our goals are to develop a new scoring system that uses easy-to-obtain and efficient variables for the prediction of the in-hospital outcome of liver cirrhosis patients.

## Materials and methods

Population and study design

This retrospective cohort study included 110 patients with liver cirrhosis who were hospitalized at Prof. Dr. I.G.N.G. Ngoerah General Hospital. Adult patients with liver cirrhosis were identified using a computerized database, covering the period from November 2021 to January 2022. The inclusion criteria were as follows: (a) patient with liver cirrhosis, (b) adults aged above 18 years, and (c) admitted to the hospital with complications of cirrhosis or emergency causes. Cirrhosis was diagnosed either clinically or through radiological methods. The diagnosis was clinically determined by identifying symptoms, such as ascites, jaundice, encephalopathy, variceal bleeding, splenomegaly, and the presence of spider angiomas. Radiological diagnosis relied on findings such as liver tissue inhomogeneity, liver parenchymal nodules, ascites, splenomegaly on ultrasonography, or a METAVIR score of F4. The exclusion criteria were as follows: (a) admission for a biopsy, endoscopy, or other elective procedure and (b) incomplete records.

Demographic, clinical, and laboratory parameters of the study

A total of 23 variables were documented upon admission. Demographic data included age and gender, with age categorized into two groups: ≥60 years and <60 years. Clinical symptoms at admission included the presence of ascites, hematemesis/melena, or hepatic encephalopathy. Comorbidities, including diabetes mellitus type II, acute kidney injury, and chronic kidney disease, were documented. Spontaneous bacterial peritonitis was diagnosed per protocol. The etiology of liver cirrhosis was identified through serological testing. Hepatitis B infection was indicated by the presence of HBsAg, while hepatitis C was defined by reactive anti-HCV. Cases not attributed to hepatitis B or C were classified as unspecified non-B non-C (NBNC). Routine laboratory tests were obtained from the medical records. Leukocytosis was classified as a leukocyte count of ≥11 x 10³/μL, anemia was indicated by a hemoglobin level of <10 g/dL, and thrombocytopenia was identified by a platelet count of <150 x 10³/μL. Serum AST and ALT levels were categorized as either normal or abnormal if they were more than twice the upper limit of normal. Abnormal bilirubin levels were defined as total bilirubin exceeding 1.2 mg/dL and direct bilirubin exceeding 0.5 mg/dL. A normal INR range was established as 0.9 to 1.1, while aPTT was considered normal if it fell between 24 and 36 seconds; values outside these ranges were classified as abnormal. Hypoalbuminemia was defined as an albumin level below 3.5 g/dL, and a high BUN level was indicated by values greater than 23 mg/dL.

We also collected the cause of in-hospital mortality based on the International Classification of Diseases, Tenth Revision (ICD-10) coding system. The cause of death was obtained from the death certificate and electronic medical record in the hospital.

Statistical analysis

Statistical analysis was carried out using Statistical Product and Service Solutions (SPSS, version 21.0; IBM SPSS Statistics for Windows, Armonk, NY). Continuous variables are presented as mean ± standard deviation for normally distributed data; for data that are not normally distributed, values are shown as median (minimum-maximum). Comparisons were made using either an independent T-test or a Mann-Whitney U-test, based on the nature of the data. Categorical variables were reported as frequencies. Bivariate analysis was performed using either the chi-square test or Fisher's exact test. Variables with a p-value of <0.25 were considered for inclusion in the multivariate analysis.

For the multivariate analysis, logistic regression was conducted using the backward LR method. All variables that were statistically significant (p<0.05) in the final model were incorporated into the scoring system. The effectiveness of the formula was assessed using the Hosmer and Lemeshow test for calibration, along with the area under the curve (AUC) for evaluating discrimination. Scores were computed using the B/SE/lowest B/SE formula. The total score probability was derived from the formula 1/1+exp(-y). To establish the optimal cutoff for the scoring system, analysis was performed using the receiver operating characteristic (ROC) curve.

Ethical clearance

This study received approval from the Research Committee of the Faculty of Medicine at Udayana University/Prof. Dr. I.G.N.G. Ngoerah General Hospital, under the Ethical Clearance Number: 1739/UN14.2.2VII.14/LT/2024.

## Results

Among the 110 patients admitted with liver cirrhosis at Prof. Dr. I.G.N.G. Ngoerah General Hospital, were included, 52 (47.3%) died during hospitalization. The median age of the patients was 54.5 years (30-85), and 33 patients (30%) were elders above 60 years old. Patient characteristics are presented in Table 1.

**Table 1:** Characteristics of liver cirrhosis patients M:F (male:female), HBV (hepatitis B virus), HCV (hepatitis C virus), NBNC (non-B non-C hepatitis virus), AST (aspartate transaminase), ALT (alanine transaminase), aPTT (activated partial thromboplastin time), INR (international normalized ratio), BUN (blood urea nitrogen) *independent samples t-test, **Mann-Whitney U-test, †Chi-square

Variables	All subject (n=110)	Died (n=52)	Survive (n=58)	P value
Age, median (min-max)	54.5 (30-85)	57 (31-81)	53 (30-85)	0.244
Gender (M:F)	(80:30)	(44:9)	(36:21)	0.091
Comorbidity				
Diabetes mellitus	17 (15.5)	12 (22.6)	5 (8.8)	0.044
Chronic kidney disease	13 (11.8)	8 (15.1)	5 (8.8)	0.305
Acute kidney disease	15 (13.6)	13 (24.5)	2 (3.5)	0.001^†^
Etiology				
HBV	56 (50.9)	31 (58.5)	25 (43.9)	0.008^†^
HCV	27 (24.5)	6 (11.3)	21 (36.8)	
NBNC	27 (24.5)	16 (30.2)	11 (19.3)	
Child-Pugh score, median (min-max)	9.5 (5-14)	11 (5-14)	8 (5-13)	0.000**
Child-Pugh class, n (%)				
A	14 (12.7)	2 (3.8)	12 (21.1)	
B	41 (37.3)	13 (24.5)	28 (49.1)	
C	55 (50.0)	38 (71.7)	17 (29.8)	
Laboratory parameter				
AST, U/L	64.25 (0.0-1497)	101.0 (0.0-1497)	46.5 (12.1-723.5)	0.000**
ALT, U/L	37 (6.9-944)	62.0 (6.9-944)	26.1 (7.0-404.6)	0.000**
Hemoglobin, g/dL	9.4 ± 3.08	9.8 ± 2.57	9.0 ± 3.46	0.171
Platelet, x 10^3^/mm^3^	125.5 (1.0-551)	140 (1.0-463)	113 (14.0-551.0)	0.219
Leukocytes, 10^3^/mm^3^	8.4 (1.59-34.88)	10.9 (2.55-34.37)	6.67 (1.59 (34.88)	0.000**
Albumin, g/dL	2.59±0.76	2.36 ± 0.72	2.80 ± 0.74	0.002*
Total bilirubin, mg/dL	2.8 (0.17-36.3)	3.5 (0.4-36.3)	1.8 (0.17-14.70)	0.000**
Direct bilirubin, mg/dL	1.34 (0.09-23.74)	2.18 (0.27-23.47)	0.99 (0.09-10.01)	0.000**
aPTT, seconds	34.3 (22.5-99.8)	36.7 (22.5-99.8)	32.2 (23-56.8)	0.002**
INR	1.4 (0.85-6.4)	1.6 (0.85-6.40)	1.24 (0.94-2.14)	0.001**
BUN, mg/dL	21.6 (3-124.7)	25.9 (4.5-124.7)	18.3 (3.0-85.5)	0.001**
Creatinine, mg/dL	0.96 (0.51-9.41)	1.1 (0.51-9.41)	0.85 (0.57-2.51)	0.003**
Cause of first hospitalization				
Encephalopathy hepatic				
No	62 (56.4)	16 (30.2)	46 (80.7)	0.000^†^
Grade 1-2	34 (30.9)	25 (47.1)	11 (15.8)	
Grade 3-4	14 (12.7)	12 (22.7)	2 (3.5)	
Ascites				
No	40 (36.4)	9 (17.0)	31 (54.4)	0.000^†^
Grade 1	16 (14.5)	9 (17.0)	7 (12.3)	
Grade 2	31 (28.2)	22 (41.5)	9 (15.8)	
Grade 3	23 (20.9)	13 (24.5)	10 (17.5)	
Hematemesis/melena	46 (41.8)	19 (35.8)	27 (47.4)	0.221
Complication				
Spontaneous bacterial peritonitis	13 (11.8)	8 (15.1)	3 (5.3)	0.086

The causes of death among our population are shown in Table 2. The cause of death varied, the most common causes being multiple organ failure (32.1%), septic shock (22.6%), and massive bleeding (22.6%).

**Table 2:** Cause of death in deceased patients

Cause of death in 52 liver cirrhosis patients, n (%)	
Multiple organ failure	17 (32.1)
Septic shock	12 (22.6)
Massive bleeding	12 (22.6)
Hepatic encephalopathy	6 (11.3)
Hypovolemic shock	3 (5.6)
Hospital-acquired pneumonia	2 (3.7)
Cardiogenic shock	1 (1.8)

We collected the variable that is potentially a predictor of in-hospital mortality in liver cirrhosis patients. The variables included were demographic data, clinical symptoms at admission, and routine laboratory examination. Eighteen variables were included in the bivariate analysis (Table 3). Notably, the completeness of the data for all variables considered in the statistical model analysis was 100%.

**Table 3:** The final model of multivariate analysis with logistic regression (n=110), from the total of 18 variables, along with its scoring points ALT (alanine transferase), SC (serum creatinine), INR (international normalized ratio)

Variables	B	SE	Exp (B)	P Value	95% CI		Score
					Upper	Lower	
Diabetes mellitus type II	3,083	1,044	21,813	0,003	168,779	2,819	1
Acute Kidney Injury	2,563	1,069	12,969	0,017	105,359	1,596	1
Hepatic Encephalopathy	2,511	0,790	12,316	0,001	57,946	2,617	2
Ascites	1,523	0,679	4,587	0,025	17,361	1,212	1
ALT ≥68	3,212	0,989	24,833	0,001	172,371	3,578	2
SC ≥1,25	3,456	1,363	31,698	0,011	458,729	2,190	1
Abnormal INR	1,678	0,846	2,354	0,047	28,107	1,020	1

The final model created from the discovery dataset revealed that three variables were statistically significant: the presence of (p = 0.001), ascites (p = 0.025), diabetes mellitus type II (p = 0.003), acute kidney injury (p = 0.017), ALT ≥ 68 (p = 0.001), creatinine level ≥ 1.25 (p = 0.011), and abnormal INR (p = 0.047). Table 3 presents the estimated coefficients (standard error) and odds ratios (OR), along with their 95% confidence intervals (CI) obtained from the multivariate analysis. To determine the quality of the analysis, the Hosmer and Lemeshow test indicated a p-value greater than 0.05. The area under the ROC curve (AUC) for the final model was 92.8%, with a p-value of less than 0.001. At a cutoff point of ≥ 2.5, the pilot scoring system demonstrated a sensitivity of 90.4% and a specificity of 74.1% for predicting in-hospital mortality in patients with liver cirrhosis (AUC: 0.913; SE: 0.026; p < 0.001; 95% CI: 0.862-0.964).

**Figure 1:**
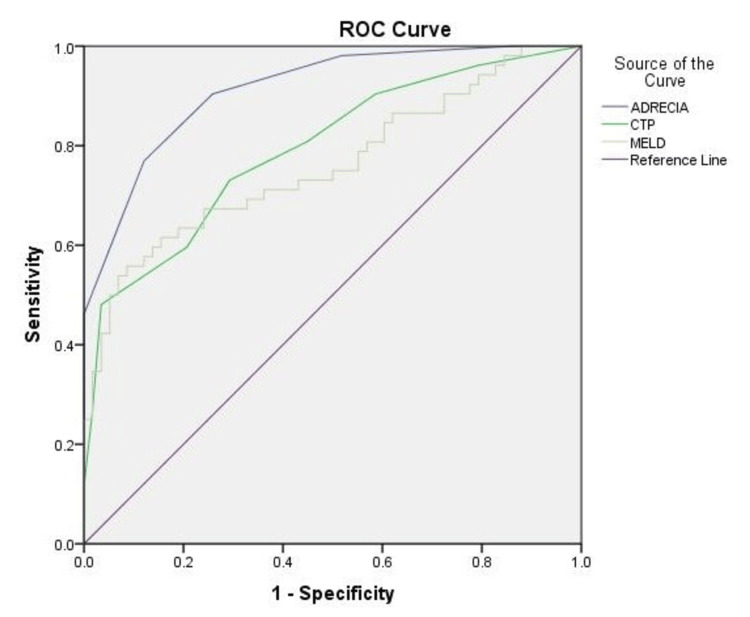
The ROC curve of the ADRECIA score (AUC: 0.91; 95%CI: 0.862-0.964), CTP score (AUC: 0.793; 95%CI: 0.709-0.877), and MELD score (AUC: 0.761; 95%CI: 0.668-0.852) ROC (receiver operating characteristic), AUC (area under the curve)

Table 4 shows the final scoring card to predict in-hospital mortality in liver cirrhosis patients. The ADRECIA score comprises ascites, type II diabetes mellitus, renal impairment or acute kidney injury, serum creatinine, abnormal INR, and elevated ALT. The probability of in-hospital mortality in liver cirrhosis patient based on their score at admission is presented in Table 5. A score of four or higher is classified as high risk for in-hospital mortality, with a probability exceeding 74.69%. Compared to the CTP score and MELD score, the ADRECIA score shows better accuracy in determining the risk of in-hospital mortality in liver cirrhosis patients (Figure 1).

**Table 4:** The final model of the new scoring system to predict in-hospital mortality of liver cirrhosis patients INR (international normalized ratio), ALT (alanine transferase)

Scoring system to predict in-hospital mortality in liver cirrhosis patients						
Patient’s name						
No				Yes	No	Patient’s score
1.	Ascites			1	0	
2.	Type II diabetes mellitus			1	0	
3.	Acute kidney injury			1	0	
4.	Hepatic encephalopathy			2	0	
5.	Creatinine serum ≥1.25			1	0	
6.	Abnormal INR			1	0	
7.	ALT ≥68			2	0	
Total						

**Table 5:** Interpretation of the ADRECIA score

Interpretation	Total Score	Probability of in-hospital mortality
Low Risk	0	1.38%
	1	5.07%
	2	16.91%
	3	43.66%
High Risk	4	74.69%
	5	91.83%
	6	97.72%
	7	99.34%
	8	99.84%
	9	99.96%

## Discussion

Determining the prognosis of the patient is a critical part of the initial evaluation of any disease. Identification of poor prognostic patients admitted with liver cirrhosis allows better risk stratification and guides clinicians to the most appropriate therapeutic planning case-by-case. Our study shows a high in-hospital mortality rate (43.7%), which is higher than previously reported in Indonesia. Nababan et al. reported that only 12.03% of cirrhosis patients died during hospitalization [10]. The current study includes patients with HCC as participants, which is attributed to the high mortality rate. Hence, a study by Nartey et al. with liver cirrhosis and HCC reported an in-hospital mortality rate of 54.1% [14], which is close to our findings.

This study presents a scoring system consisting of DM, AKI, HE, ascites, elevated ALT, increase in SC, and abnormal INR. In line with our finding, a high creatinine level was an independent predictor of in-hospital mortality. Renal dysfunction in patients with liver cirrhosis may be caused by one or a combination of factors: bleeding or hypovolemia, bacterial infection, hepatorenal syndrome, and drug use [10]. Thus, acute renal failure could be a sign of worsening conditions in patients with liver cirrhosis. Acute renal failure and prolonged prothrombin time have been identified as predictors of in-hospital mortality in cirrhosis patients with alcoholic liver disease [15].

A study by Bianchi et al. reported DM, along with albumin, ascites, age, encephalopathy, bilirubin, and platelets, was an independent prognostic factor of mortality in patients with liver cirrhosis [16]. DM significantly increases the in-hospital mortality rate in the elderly with liver cirrhosis. Poor diabetic control is associated with worsening renal function and incidence of hepatic encephalopathy [17]. Additionally, DM accelerated liver fibrosis and inflammation, leading to more severe liver failure [18,19]. Ascites is characterized by fluid accumulation intra-abdomen. Severe ascites increase the risk of spontaneous bacterial peritonitis due to bacterial translocation or dysregulation of the immune system. The presence of ascites is associated with a higher risk of mortality, with an incidence rate of 15% in the first year and 44% over the first five years [20].

Duah et al. identified hepatic encephalopathy, elevated creatinine, INR, and MELD-Na as predictors of mortality in hospitalized cirrhosis patients [21]. Hepatic encephalopathy associated with portal hypertension leads to the opening of spontaneous portosystemic shunts. Furthermore, HE also reflects other pathophysiology mechanisms, including impaired nutritional status, sarcopenia, and frailty [22]. Therefore, the development of HE is associated with poor outcomes. In-hospital mortality in patients with liver cirrhosis admitted with HE was 75.5% [21].

A study by Paul et al. showed that a longer duration of disease, high leucocyte count, high neutrophilia, higher INR, high creatinine, and high ALT are associated with mortality in patients with liver cirrhosis [23]. A coagulation dysfunction, identified at an INR of 1.6, was associated with increased mortality in liver cirrhosis patients [24]. From a pathophysiological perspective, an elevated INR indirectly indicates that patients with decompensated cirrhosis have inadequate liver function reserve. It is also a predictor of variceal bleeding, which means that elevated INR is associated with significant portal hypertension [25].

Thuluvath et al. developed a prediction model for in-hospital mortality in cirrhosis patients with hyponatremia. Their model showed an AUC of 0.80 (95%CI: 0.78-0.82), incorporating age > 65 years old, variceal bleeding, sepsis, coagulopathy, and acute-on-chronic liver failure [26]. Furthermore, Lin et al. reported a prediction model for in-hospital mortality in liver cirrhosis, which included age, heart rate, total bilirubin, glucose, sodium, anion gap, fungal infection, mechanical ventilation, and vasopressin with AUC value 0.805 (95% CI: 0.776-0.883) [27]. The previous study has reported a logistic and additive scoring system to predict in-hospital mortality in acutely decompensated liver cirrhosis in Indonesia. The scoring system consisted of age, bacterial infection, total bilirubin, and creatinine, with a discriminative point of 0.868 and 0.899 [10]. Bacterial infection as an independent predictor seems to be subjective due to different definitions center-by-center. The current study has developed a simple scoring system to predict in-hospital mortality in liver cirrhosis. Scores are calculated using clinical data that are routinely obtained at the time of hospitalization. Therefore, this scoring system can be applied at the bedside in a resource-limited clinical setting. Furthermore, our scoring system showed better accuracy compared to MELD and CTP.

Nevertheless, there are several limitations to our study. Firstly, this study was conducted at a single center with a limited sample size, which may limit the generalizability of the findings to other populations. Secondly, we performed a retrospective cohort study that led to the risk of information bias. Further validation of the scoring system for predicting in-hospital mortality in liver cirrhosis patients in different populations is needed to confirm our findings.

## Conclusions

The ADRECIA score is a novel and practical tool derived from routine clinical data to predict in-hospital mortality in patients with liver cirrhosis. It is simple, easy to use, and integrates widely available laboratory indicators, making it suitable for primary assessment. Despite its simplicity, the score demonstrates high predictive accuracy. Further studies are recommended to validate its performance across different clinical settings and populations.
